# The Origins of AIDS

**DOI:** 10.3201/eid1807.120461

**Published:** 2012-07

**Authors:** Kevin M. De Cock

**Affiliations:** Centers for Disease Control and Prevention, Atlanta, Georgia, USA

**Keywords:** HIV/AIDS and other retroviruses, AIDS, origins, HIV, book review, virus, Kinshasa, iatrogenic hypothesis, molecular epidemiology

This excellent but frustrating book is essential reading for anyone deeply interested in the early history and dissemination of HIV/AIDS. Interest must be deep because the author spares few details about colonial medical systems in the former French and Belgian territories, Congolese politics around independence, and a host of other obscure matters.

The book’s strengths include clear explanations of complex themes, such as the molecular evolution of simian and human retroviruses, and a comprehensive review of early events in the pandemic. Many of the book’s sections are engaging. For nonspecialists, the book provides some of the most intelligible analyses of molecular epidemiology and the early history of HIV/AIDS, including consideration of different explanations of the origin of HIV. For example, the author usefully examines and dismisses the hypothesis still promulgated by Edward Hooper that HIV originated in eastern Congo during the 1950s after material grown in monkey or chimpanzee cell cultures was used for mass vaccination against polio. Nonetheless, readers will cover a lot of material that could have been omitted or skip sections not essential to the core theme.

Fascinating insights and anecdotes are scattered throughout the text. The reader will find commentaries on early tropical researchers and public health officials, as well as description of a cryptic wasting illness in the 1930s referred to as “Cachexie du Mayombe.” The clinical description of patients with this syndrome is eerily reminiscent of patients with AIDS: “an assembly of bones held together by skin… whose only life lay in their gaze.” There is also an incidental but valuable discussion of the late Jonathan Mann, founding Director of the World Health Organization’s Special (later Global) Programme on AIDS, who deserves his own full historical biography.

This book represents a personal mission for Jacques Pepin, a Canadian infectious disease specialist and epidemiologist with broad African experience who developed an abiding interest in human African trypanosomiasis (sleeping sickness). Pepin’s thesis regarding HIV derived from findings from retrospective studies of HIV-2 that he then applied to HIV-1. He proposes that during the colonial era in central and western Africa, the extensive re-use of needles and syringes in medical practice and campaigns against endemic tropical diseases amplified the early spread of HIV-1 after cross-species transmission from chimpanzees. Kinshasa became the early HIV epicenter from which subsequent global dissemination occurred, principally through sexual transmission.

The evidence offered by Pepin for the iatrogenic hypothesis is probably better presented than ever before. Nevertheless, the evidence is speculative because it is based on circumstantial and ecologic associations, such as those between earlier medical practices and trends in hepatitis C virus infection. Despite excessive speculation in parts of the book, such as that concerning the role of the trade in plasma from Haiti, this work is still a “tour de force” and deserves widespread recognition.

